# An assigned responsibility system for robotic teleoperation control

**DOI:** 10.1007/s41315-018-0043-0

**Published:** 2018-01-19

**Authors:** Nicolas Small, Kevin Lee, Graham Mann

**Affiliations:** 1 imec-DistriNet, KU Leuven, Celestijnenlaan 200a, Heverlee, Belgium; 2 School of Science and Technology, Nottingham Trent University, Nottingham, UK; 3 School of Engineering and Information Technology, Murdoch University, Perth, Australia

**Keywords:** Assigned responsibility, Teleoperation, Robotic navigation

## Abstract

This paper proposes an architecture that explores a gap in the spectrum of existing strategies for robot control mode switching in adjustable autonomy. In situations where the environment is reasonably known and/or predictable, pre-planning these control changes could relieve robot operators of the additional task of deciding when and how to switch. Such a strategy provides a clear division of labour between the automation and the human operator(s) before the job even begins, allowing for individual responsibilities to be known ahead of time, limiting confusion and allowing rest breaks to be planned. Assigned Responsibility is a new form of adjustable autonomy-based teleoperation that allows the selective inclusion of automated control elements at key stages of a robot operation plan’s execution. Progression through these stages is controlled by automatic goal accomplishment tracking. An implementation is evaluated through engineering tests and a usability study, demonstrating the viability of this approach and offering insight into its potential applications.

## Introduction

The control of robots is a non-trivial task requiring complex perceptions, decisions and actions to be made using limited information. While many teleoperated systems still only employ human operators, the inclusion of automation is now much more common. Automated control rarely completely replaces human operators, because contemporary technology is not yet adequate, but automation can support their work. Mixing of control shows much promise, relieving operators of workload, and allowing the use of teleoperated robots in environments where they could not be previously controlled (on the surface of Mars for example McCurdy 2009). Adjustable and adaptable autonomy systems allow the level of automation to be raised or lowered during task execution. This allows the capabilities of both the human operator and the automation to be appropriately paired to the sections of the overall task they suit best.

Mixing human and automated control of a system is not without a cost. There are well understood problems with integrating humans in automated control systems Parasuraman and Riley (1997). Such systems introduce additional complexity by offering a variety of ways the human operators can interact with the automation, compounding these integration issues and introducing extra engineering challenges. Indeed, systems have to be able to manage the reconfiguration of control including both the provision of a range of different modes or levels of automation (LoA), and the ability to select among them for a given situation. A broad spectrum of ten such levels, ranging from all but full human control to total automation was initially introduced by Sheridan and Verplank Sheridan and Verplanck (1978); at the time, only the more human-controlled levels could practically be implemented. Based on the notion that human–machine control had four implicit subtasks (monitoring, generation of options, selection and execution of an option), Endsley (1999) subsequently offered a more specific set of ten levels that detailed how these subtasks would be assigned and managed in each case. This scheme has since enjoyed considerable adoption, either in part or whole, by experimental systems Prewett et al. (2010) but for particular experimental systems or task requirements, a narrower range customised control modes is sometimes used (e.g. Wang and Lewis 2007).

Setting the LoA is a *meta-control task*Goodrich et al. (2001) that could itself be done either by the human operator, automatically, or be a shared responsibility. Most systems rely on the human operator to trigger changes in levels (Muszynski et al. 2012; Stentz et al. 2015; Yi et al. 2015; Kohlbrecher et al. 2015); others automate that process (Sellner et al. 2006; Martinez-Tenor and Fernandez-Madrigal 2015). Still others use a mix of automated and human decision-making (Cote et al. 2012; Hardin 2009). When the human operator alone is responsible for switching, the extra load on the operator’s cognitive resources amounts to those perceptions and actions needed to make the switching decision. An automated switching strategy relieves some but not all of this extra burden; the operator must still monitor these dynamic changes and be prepared for manual operation at any time. In mixed control modes, the operator must take on all of these loads, plus the additional overhead of deciding if he/she should take over a control decision. These demands are considerable and the factors affecting them are not simple (for a review of factors affecting HRI workload, see Prewett et al. 2010). Furthermore, whether or not that change is planned is likely to have an impact on performance. For example, a system that automatically adjusts its level of autonomy must be able to cope with the human operator not being available or ready to take over, either through reaching out to the operator via messaging if there is an operator “on call”, or by settling into a safe state until someone can attend to it. That complication could be avoided in a system with planned changes.

Even in situations where there is a clear candidate for the most effective LoA for the given task elements, existing systems do not involve planned changes; rather this is left to the decision-making agent at runtime. This allows for flexibility in the face of unexpected events, but it will be argued here that in situations where the task can be pre-planned, much can be gained by trading off some of that flexibility.

Assigned responsibility (AR), is a type of adjustable autonomy that we initially proposed in Small et al. (2015) to allow this. This paper expands on these ideas, and proposes, implements, and evaluates an architecture for Assigned Responsibility. This approach, unlike previous adjustable autonomy systems, pre-plans changes in the level of robot autonomy and triggers those changes automatically at runtime to: (1) relieve operators of the additional task of effecting those changes, (2) reduce operator confusion with regards to responsibility in each step by allowing this responsibility to be known ahead of time, (3) allow breaks to be planned during steps that don’t require one or more operators, and (4) allow the more aggressive automating of a task by keeping each step small and self contained. This paper presents a prototype implementation of an Assigned Responsibility system, establishes the feasibility of the architecture, and evaluates the system’s performance and its positive impacts on the operator’s workload in a series of experiments using a real robot.

The remainder of the paper is structured as follows. Section 2 justifies the creation of the new model and details the basic principles of an AR system. Section 3 describes a proposed architecture for AR. Section 4 presents a practical implementation of AR in a teleoperated robot. Section 5 presents the experimental evaluation of the AR robot in a model maintenance task. Section 6 presents the observations and discusses future work. Finally, Section 7 presents some conclusions.

## Principles of assigned responsibility

In common with other approaches to teleoperation, the motivation behind the AR approach is to improve system performance (increase effectiveness and efficiency, reducing errors and task completion times) while improving operator experience (reducing cognitive load, stress and fatigue) by designing better hardware and software for the whole system. A particular focus of this work is a special management of automatic LoA switching, reduction of operator confusion and fatigue, and support for the gradual automation of the execution of the full task over time. The concept is based on five observations, or matters of emphasis, which form the core principles of the design:

1. *In some domains, real robot operations can be well-planned in advance.* Whether to direct and regulate human workers, autonomous robots or humans using robots, operations plans are currently used in much existing work. In relatively predictable environments such as warehouses (Crosby and Petrick 2014) and farm fields (Zion et al. 2014), plans are effective in controlling robot tasks, while in tasks that involve complexity, danger to human workers, inconvenience to the public, or great expense to stakeholder organisations, such as oil rig maintenance (Nas et al. 2009) and building construction (Hendrickson and Au 1989), operations plans are already well established practice. The plans are usually prepared manually but today planning algorithms implemented in software toolsmay be used to assist (McCurdy 2009; Ding et al. 2013).
2. *In mixed control situations, the risk of operator confusion, or unpreparedness, is ever-present.* The problems of loss of situational awareness and perceptual and control skills (the *human-out-of the loop problem*) has long been understood to be a source of trouble in HRI (Endsley and Kiris 1995). Machine operator control conflicts in mixed control modes have been implicated in accidents and inefficiency in several cases (Sheridan and Parasuraman 2005). That a driver could be caught unprepared to suddenly take over control in an autonomous vehicle is now a serious concern for designers of these systems (Ohn-Bar and Trivedi 2016). All this suggests that human–machine interactions could be improved by communicating control responsibilities as clearly as possible at every turn. Previous work has suggested that a method to avoid the human-out-of the loop problem is to ensure the operator is implicated in more of the work being done (i.e.: generally operating at a lower LoA, Kaber et al. 2000). To address the human-out-of the loop problem without increasing operator workload, AR instead offers a contractual method of ensuring that operational responsibilities are clearly designated and fully understood by the human operator. This ensures an operator returning from a break is presented with the context of the current task: i.e.: what has happened so far and what is yet to happen.
3. *The resilience of teleoperated machines to neglect—how quickly and badly robot performance degrades when human control ceases—should be improved by better design.* Since Goodrich et al. (2001) conceptualised the effect of LoA on a system’s tolerance of neglect, moderating the degradation in performance of a system that was not or could not be attended by a human controller has been seen as an opportunity for mixed automation. Apart from wasted time, an AR robot shifting from a highly automated LoA to a human-dependent level will simply wait and generally not lose performance if the human operator does not take control, unless the task has left the system in an unsafe state. Task plans can be designed not to do that; automated modules can notice unstable conditions and append safeing actions to the end of the automated task by making stability a condition of goal satisfaction. Yet the full benefits of neglect for *operators* does not seem to have been appreciated. There are good reasons to think that a well designed system could do better than simply degrade gracefully. Planned shifts in LoA could potentially be used to better manage mode transitions such as by the use of messages to anticipate the imminent need for operators to take control, or provide timing for permissible breaks. The authors know of no other design that would encourage operators to relax their vigilance of the system temporarily.
4. *Operator workload, fatigue and frustration could be reduced by planned but automatic changes in LoA at conspicuous task boundaries.* Reducing operator fatigue and operator errors is of prime importance to the designing of teleoperation interfaces (Fischer et al. 1990), and therefore needs to be a driving factor in the design of new paradigms such as AR. When progress through a work plan is automatically tracked (Small et al. 2013), the edges of the hierarchical graph, representing transition points in task control requirements, become conspicuous. We argue (below) that these transition points are convenient places to plan shifts in LoA. It is hypothesised that the automatic tracking, automatic LoA switching and possible task automation will strike the right balance between human understanding and automated efficiency. The experiment described in Sect. 5 concerns this use of an explicit work-sharing ‘contract’ between the operator and the robot about roles to reduce confusion, ease operator workload and fatigue and improve speed and error rates.
5. *Plans subdivide work into a hierarchy of tasks, providing opportunities that benefit the development of more automation over time.* Assuming that the ultimate goal is much more complete coverage of tasks by automation, consider the question of whether an AR system could help with its own development. We believe that software developers could be assisted in building and testing automated modules. First, because the operation is partitioned into labelled control tasks, statistics on the performance of the human–machine system can be meaningfully recorded for each. For example, for a given task performed in a particular level, the duration, number of errors, and other performance metrics can be tabulated for each execution. Subjective measures of stress and frustration could even be elicited from the operator through the interface after the goal was accomplished in a task. These data could then help create development targets for new automated modules, and signal problems with existing ones. Second, a human expert teleoperating a robot in a work context offers the prospect of capturing valuable exemplar data that could be used by a machine learning program to duplicate the skilled operations autonomously. Although such Learning by demonstration is not easy (Argall et al. 2009; Ge et al. 2014), being able to collect the needed human data relatively effortlessly in the course of normal operations is advantageous. Furthermore, additional goal information that can aid learning is also readily available in an AR system. Partitioning exemplar data according to clearly articulated goals can help avoid perceptual aliasing, which otherwise troubles this kind of machine learning (Pastor et al. 2009; Chao et al. 2011; Manschitz et al. 2015).

The landscape of existing strategies for control switching found in the literature has at least two dimensions: who is responsible for changes (human, automation or mixed) and how changes are triggered (in a planned and predictable manner, or dynamically in response to environmental and task factors, or some combination of both). The responsibility for change is a matter of design. Adjustable autonomy systems (Goodrich et al. 2001; Stentz et al. 2015), depend on the human operator and tend to use occasional automatic intervention to relieve that human operator of workload, allowing some respite. Adaptive autonomy systems, on the other hand (Cote et al. 2012; Sellner et al. 2006), favour autonomous operation when possible, reverting to partial or total human control when the automation fails. In these systems, control over LoA is at least partially automated. Mixed initiative systems use both in a variety of combinations.

**Fig. 1:**
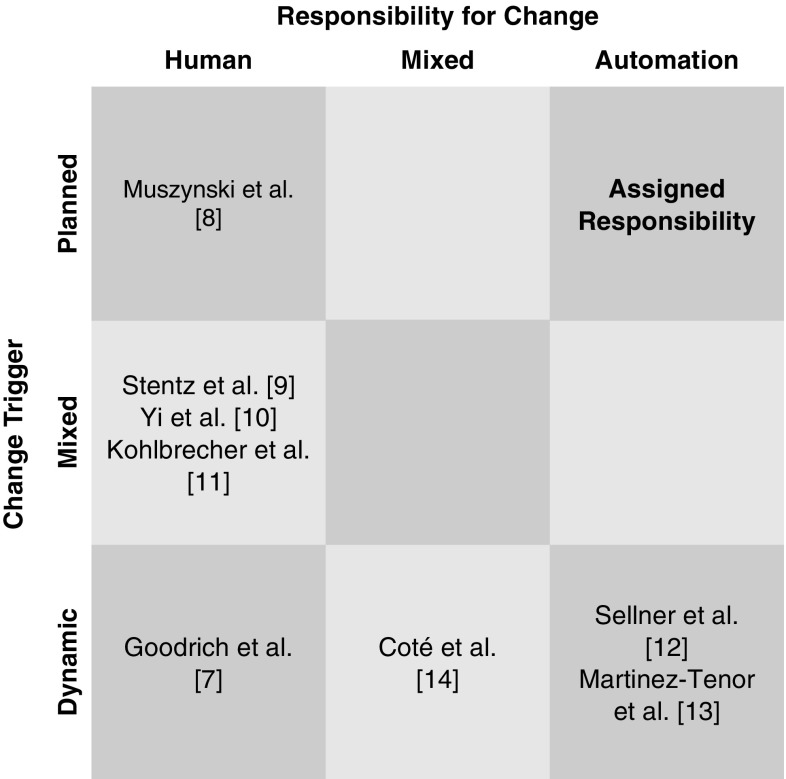
Levels of autonomy changes in AR and other control systems

The other dimension of interest here is whether these changes are planned (e.g. when doing a given task the system is set to a particular mode), or dynamic (e.g. changes in levels which occur as a response to environmental changes). The distinction is not always clear cut, because a system with planned changes still needs to be able to dynamically respond to errors, but the normal operating procedure for the system is likely to rely on one of these change strategies more than the other. Figure 1 organises the meta-control strategies of several systems into a single diagram, suggesting that that extant automation-centred adjustable autonomy systems tend to rely on dynamic level changes, as the automation reacts to handle situations beyond its capabilities, while human-centred adjustable autonomy systems might use any kind of trigger.

None of these systems use planned, automatic changes in LoA. Miller and Parasuraman (2007), p. 63 discuss the possibility of a system organised in this way, but their delegation architecture does not correspond to our design). This however seems like a logical step where advanced planning is possible. For example, CHIMP (Stentz et al. 2015), a robot taking part in the DARPA Robotics Challenge, was to perform a specific set of tasks, and could practice performing these ahead of time. During this practice, the LoA best suited to each challenge would have been found and this level would have been used during the trials. Rather than having the operator select this best LoA during the trials, the appropriate levels for parts of the job could be set ahead of execution time. To do this, AR relies on pre-planning the execution of a task by a human manager or operator, who assigns subsections of that task to either human or automated controllers. This clear, pre-determined separation of roles is key to avoiding conflicts and confusions in an environment with several operators.

AR can be understood as a scaffolding for a pre-planned adjustable autonomy teleoperation interface. It allows the LoA of a particular robot to change systematically during the execution of an overall plan. This is new because, unlike other adjustable autonomy approaches, these changes occur automatically at specific plan execution points. Figure 1 illustrates where Assigned Responsibility sits relative to other systems. AR requires a job to be broken down into steps before its accomplishment, with each of these steps assigned an autonomy level. During execution, the system then keeps track of progress through the work, automatically adjusting the LoA as specified when each step is accomplished.

By having level changes pre-planned and executed automatically, AR necessarily sacrifices some flexibility in the face of changing conditions. This exchange is motivated by four considerations: (1) A predictable schedule of responsibility allows human operators to know ahead of time when they are and when they are not needed, allowing them to plan breaks. (2) The pre-set levels of autonomy should reduce conflicts between operators, as their role at each stage is well-known to them ahead of time. (3) Pre-determined changes provide boundaries for automation to operate in, reducing programming complexity, and facilitating the gradual automation of the whole task. (4) In scenarios where the use of automation is restricted due to laws, ethical considerations, or simple mistrust in the capabilities of that automation, the ability to specify what is and what isn’t automated becomes desirable.

The flexibility issues discussed in the previous paragraph are most apparent if the plan is treated as a static object once the task is underway. Automated planning techniques (such as those described in Lee et al. 2009) can be used to mitigate this problem: if the controller reports an error (or the sensor readings indicate the occurrence of one), partial or full replanning of the plan tree can be done to fix the error, circumvent the problem, or safely return home if the plan must be abandoned.

## Architecture for assigned responsibility

This section describes an architecture to enable AR in robot teleoperation systems. The architecture is general, and not aimed at supporting any particular robot teleoperation system. It is able to be retro-fitted onto existing robot teleoperation systems. It does not intercept the operator/robot control loop, but rather sits on top of the basic architecture, communicating with the operator’s station, and observing the robot. This base system should possess the ability to operate in several different autonomy levels to make full use of AR, although systems that only operate in one level are supported. The core of the proposed architecture focuses on the *management module*, a self-contained block in charge of ensuring the LoA always set as specified before a task starts. This is performed through the operations of two main processes, *accomplishment monitoring*, and *plan management*.

### Plans as trees, and goal breakdown

To enable AR, tasks must be segmented. The proposed architecture does this by decomposing jobs into a series of goals, called *the plan*. In many domains (e.g. automated planning), a plan is represented as a graph tree in which the nodes are the goals (the individual steps), the edges between them are actions and goals are defined as desirable states of the world (Ghallab et al. 2004). Achieving a goal amounts to choosing and then performing actions that are required to change the state of the world from an undesirable state to the desirable state specified by the goal. The plan is a tree of interdependent goals, with each parent goal relying on the fulfilment of all its child nodes. The job is accomplished when the root node (the highest-level goal) is satisfied. Tree representations are useful for AR because they are easily describable using graph theory, which provides useful rules to read and understand these graphs automatically. The plan tree graph also has the advantage of being very human-readable, trees being a natural way for people to break down tasks (Sacerdoti 1977). This helps the human operator to be very clear about the plan.

To allocate tasks to either the human operator or automated control system, it is necessary to break down the top level goal into sub-goals, and those sub-goals into further sub-goals until a satisfactory goal granularity is obtained.^1^

**Fig. 2:**
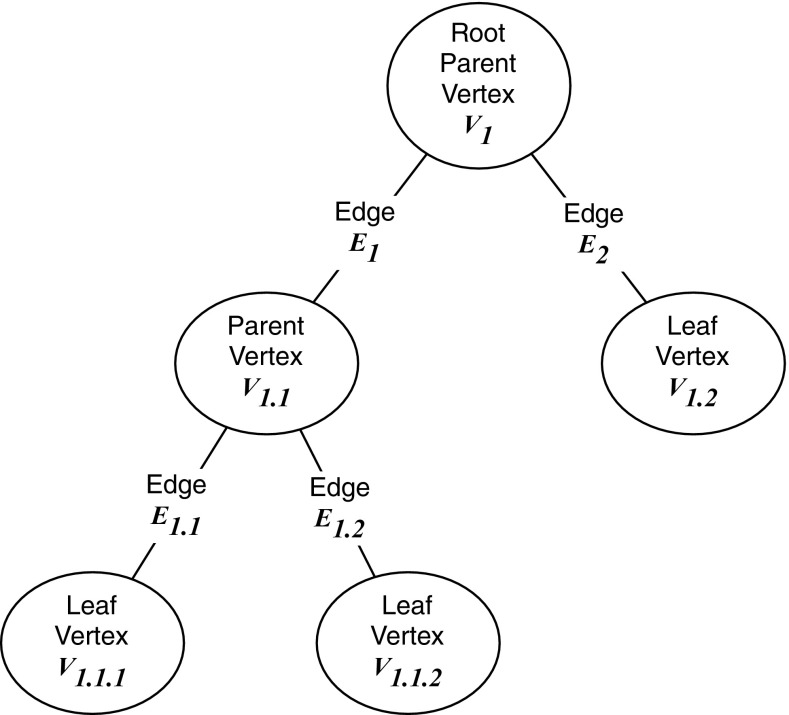
Sample ordered rooted tree. This diagram illustrates the different components a plan graph can possess: root, parent and leaf vertices, all connected by edges. The vertices are ordered according to their numbering

The resulting plan graph possesses the properties of an ordered rooted tree, the details of which are described below, and illustrated in Fig. 2.

- It can be defined as a connected acyclic graph *G* with $G = (V, E)$ where *V* is a collection of *n* vertices and *E* is the collection of $n-1$ edges.
- The vertex $(V_1)$ is designated as the root of G.
- The parent of a vertex is the vertex connected to it on the path to the root; every vertex except the root has a unique parent.
- A child of a vertex $V_n$ is a vertex of which $V_n$ is the parent.
- An ordering is specified for the children of each vertex.
- A terminal vertex (or leaf) of a tree is a vertex of degree 1.

We can interpret these theoretical concepts as follows:

- The root is the overall goal to achieve, each other vertex is a sub-goal.
- To achieve a particular sub-goal, its children have to be satisfied, in order (by convention, left to right).
- By extension, once all sub-goals are satisfied, the root goal is also satisfied.
- The leaves are the lowest-level goals, and are the only goals satisfiable directly. Satisfying all of the leaf goals, in order, satisfies the root goal.

### Accomplishment monitoring

To allow automated changes in levels of autonomy to happen as planned, an AR system must be able to track progress through the execution of a task to completion.

Current research interest in the monitoring of the execution of plans is focused on supporting the improvement of this execution, and the improvement of the monitoring itself (Lee et al. 2009). These improvements are initially motivated by the specific domains they originate from, but tend to be generalised later if the ideas are not domain-specific. For example, in the business domain, languages such as BPEL (Business Process Execution Language), seek to describe the execution of business processes in order to provide some process management capabilities, such as lifecycle management, failure recovery, and a variety of control regimes, while mostly ignoring the data being transferred between those processes (Leymann et al. 2017). Scientific workflow processing, such as is supported by workflow management engines (Deelman et al. 2004) is driven by the need to operate on large data sets (Sonntag et al. 2010), therefore placing more importance on the data flowing between processes. Regardless of low-level differences, these systems provide similar high-level plan execution monitoring functionality, and seek to solve similar problems at that level.

To track the progress of a plan’s execution, a monitoring system needs to be able to actively fetch or wait for an update on goal statuses during execution (Small et al. 2013). BPEL does this through web service interfaces, whilst scientific workflow management systems commonly utilise log file parsing. After the data is collected, it needs to be analysed for patterns, simple and complex, that indicate the current status of plan execution. Depending on the state of the plan execution, it can continue, or be re-planned. This process has been formalised by the Autonomic Computing community to support adaptive systems (Kephart and Chess 2003).

The segmentation of tasks into plans of goals brings along advantages for accomplishment monitoring in AR systems. It is convenient to represent goals in the same form as observed states of the world as provided by sensors. Checking the accomplishment of a goal is then as simple as comparing it with a current set of world-state representations as shown in Small et al. (2013). For example, if a robot is equipped with a sensor which returns its position in two-dimensional space, a locational goal might be specified as ‘robot_at(250, 300)’. This can be directly compared to the output of the sensor, which might return ‘robot_at(240, 300)’. A more sophisticated arrangement would involve abstracting the goal to ‘robot_at(waypoint)’ and providing additional knowledge defining the waypoint in terms of a number of sensory tests and satisfaction conditions with respect to those tests. In this case, the waypoint is associated with a set of GPS coordinates of an area within which the target is found, a specific barcode known to be present at the target, or an image pattern to be matched against a known landmark through the use of an on-board camera. The satisfaction condition might be that the current GPS coordinates must be within bounds, and that a match on either of the other sensory indicators would be sufficient. Such straightforward arrangements would not always be enough, because the relationship between sensory data and actual world states is not always simple. Not only does the sensitivity, range and signal-to-noise ratio characteristics of a given sensor affect the interpretation of its signals, but the satisfaction or failure of some goals might involve a subtle alteration in sensed properties, possibly including necessary state progressions or alternatives. Some of the literature on robot perception deals with the control of uncertainty introduced by these complications (Thrun 2000; Minguez and Montano 2005).

Not all goals are the same, but may have different natures based on their objective and relation to processing. The proposed architecture supports three types of goals previously described in literature:

- **Achieve goals** Dastani et al. (2006) are simple expressions denoting a desired world state to be reached.
- **Maintain goals** Dastani et al. (2006) are goals that need to be protected (i.e. their accomplishment tests must not be allowed to fail). Maintenance goals require extra monitoring after they have been initially accomplished, placing an extra burden on the monitoring system.
- **Opportunistic goals** Small et al. (2013) are goals associated with a watch for particular events or world-states, the presence of which is considered favourable. Opportunistic goals mirror maintain goals, in that rather than demand checks for threats to goals, they encourage checks for contingencies favourable to goal accomplishment.

These goal types require different monitoring support. Achieve goals depend upon matching the required world-state to the current state of the world. Maintain goals seek to actively protect a desired state. This requires tests sensitive to boundary conditions around the goal state which suggest a threat, requiring protective actions to be executed. These safeguards can be included the hierarchical plan graph as special annotations. Opportunistic goals must use tests to detect occurrences known to promote the accomplishment of goals, as well as the appropriate actions (such as skipping ahead) which may be similarly included in the plan.

### Plan management

The plan management module is charged with ensuring that all of the components of the AR system are aware of the current state of the task’s achievement. This state record is used to synchronise all of these components, ensuring the LoA of the system is set as planned, the human operator is aware of the current goal, and that the accomplishment monitoring process is checking for the accomplishment of the correct goal. The plan manager relies on the accomplishment monitoring process for updates to the task progress, and is charged with integrating and disseminating those updates to trigger appropriate changes to the human–machine system.

**Table 1:** Levels of autonomy for assigned responsibility

Mode	Full name	Description
H	Full teleoperation	The human operator controls every aspect of the teleoperation process
H/A	Assisted teleoperation	The human operator controls the teleoperation process, but is assisted by simple automated systems. For example inverse kinematic control of a robotic arm
A/H	Human assisted automation	The automatic controller executes low-level robot controls. The human operator provides high-level help such as designating waypoints in navigation tasks, and object location in manipulation tasks
A	Full automation	The automatic controller has full control

### Example responsibility assignment strategy

By applying the general theories put forward by (Miller and Parasuraman 2003; Parasuraman and Miller 2006; Miller and Parasuraman 2007) to the domain of teleoperation, and Sheridan and Verplanck (1978) scale, a set of four autonomy levels was created, as described in Table 1. It is important to AR is not tied to any specific levels; these levels were simply convenient for this implementation.

In AR each of the plan’s sub-goals are explicitly assigned one of these levels before the execution of the plan. In this example assignment strategy, it is assumed that the human operator is capable of effectively controlling the robot and satisfying all of the goals using it, and that the preference is to relinquish most of the control to the automated modes in order to free up the human operator. These assumptions are for explanatory purposes only and are not, of course, taken for granted. An example assignment process is described below. This process would not typically be done by the operator, rather by a manager or robot specialist, in which case making the operator aware of the contents of the plan is a critical step.

1. All of the goals are set to full teleoperation.
2. Each leaf goal known to be at least partially automatable is set to the appropriate level. As the automatic software is programmed to accomplish more goals, more and more of the leaf goals may be assigned automated levels.
3. The resulting list of goal and associated assignments is shown to the human operator (if needed).
4. The final assignment is set, ensuring both the human operator and the automated control system are aware of the goals for which they are responsible.

### A modular design for the assigned responsibility-based control of a mobile robot

**Fig. 3:**
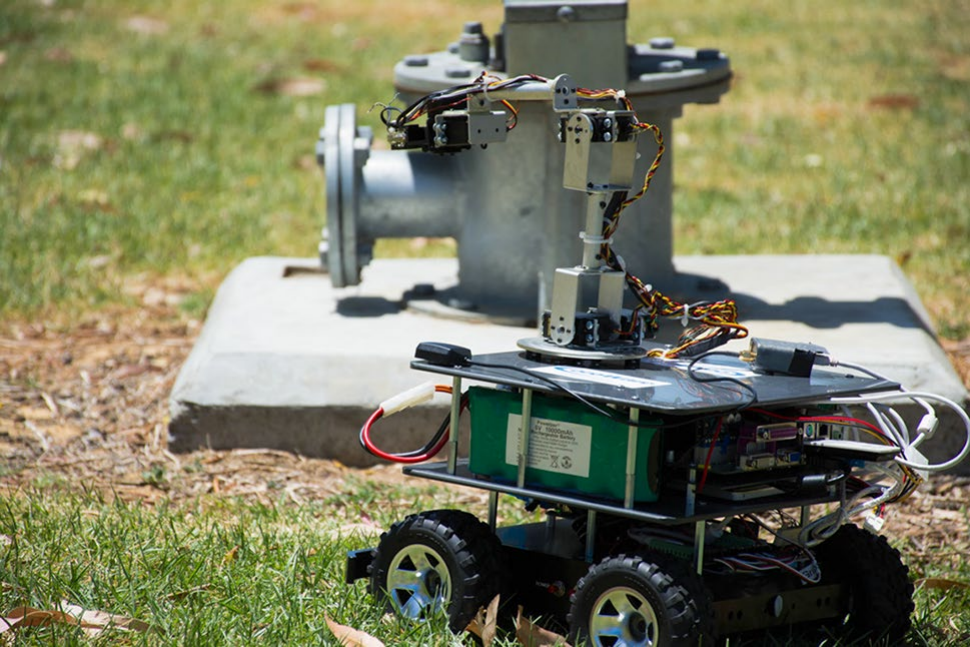
The commercial Coroware robot modified for this project approaching a work site for inspection

Automatic maintenance of physical equipment requires a mobile robot to periodically visit a number of worksites. The robot may perform tasks such as photography, gathering sensor data on environmental conditions, physically probe the integrity of surfaces, joints or attachments, remove panels and/or to change out faulty components (Mann 2008). To perform such tasks, a robot needs to be guided around multiple worksites (Fig. 3), aligning itself close to each one in turn so as to be able to address important objects.

This section describes a modular design for an AR-based control system for a small mobile robot, allowing that robot to perform the task described above under human and automated supervision.

#### A modular approach

In the maintenance scenario described above—and in most robotics applications—a robotic control system must operate over a network; at the minimum the robot itself and a control station. A modular or component-based approach is ideal when designing software that will be distributed over multiple hardware platforms connected over a network. In a modular design, individual components should be interchangeable with other components capable of performing similar tasks. This is particularly important in robotics, where a system might want to control another robot, or use a different planning algorithm, or in the case of goal accomplishment tracking, load up another goal test.

For an AR system, the most important reason to choose a modular approach is the drive to progressively automate the execution of tasks. As automation for more tasks is written, it will be added to the overall system. The easiest way to allow this to happen is to make the automated controller modular: when new automation techniques become available, modules implementing them can be created. Adding new automation capabilities to the system then simply becomes a case of making these new modules available to be loaded up during runtime. Performance statistics of each module can be accumulated once deployed. As mentioned in Section II, one of the advantages of having humans controlling the robot during non-automated tasks is that data can be collected on the human accomplishment of these tasks, possibly paving the way for future automation.

#### Design for an assigned responsibility teleoperation system

**Fig. 4:**
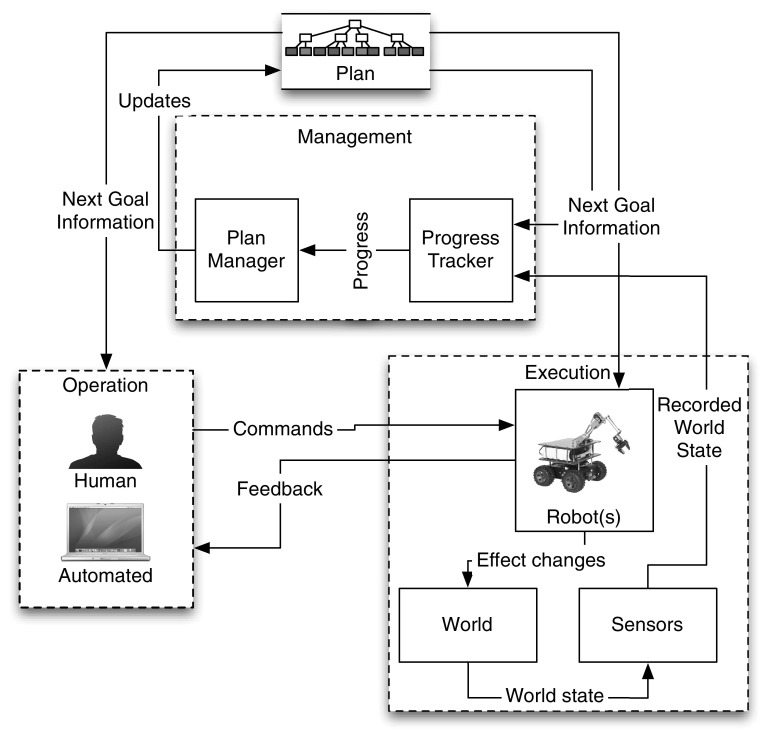
Proposed design for an assigned responsibility system

This design consists of components divided into three groups: operation, execution, and management (as shown in Fig. 4). The operation and execution groups include the basic teleoperation loop of operator sending commands to the robot and the robot executing them as well as sending feedback to the operator. The management group is concerned with tasks specific to AR. These groups are described in detail below.

**Operation** The operation group provides an abstraction layer for the command side of the remote control. Any changes in operators, or use of multiple operators should be handled within that group. The operation group contains the following modules: the user interface for the system, the automation modules, and the modules handling mixed input modes (when human and automated operators control the robot together).

**Execution** The execution group is mostly concerned with executing commands sent by the operation group, and with handling sensors mounted on the robot. It includes the main control loop for the robot, as well as an interface to handle requests for information gathered by the robot (mostly sensor data).

**Management** The management group is in charge of two major tasks: plan management, and progress tracking. The plan management part is tasked with managing the file storing the plan, making the plan and its goals available to modules that request it, and updating the plan with new information (e.g. goal accomplishment). The progress tracker monitors the plan’s execution in the real world using sensor data to keep the plan up to date.

**Fig. 5:**
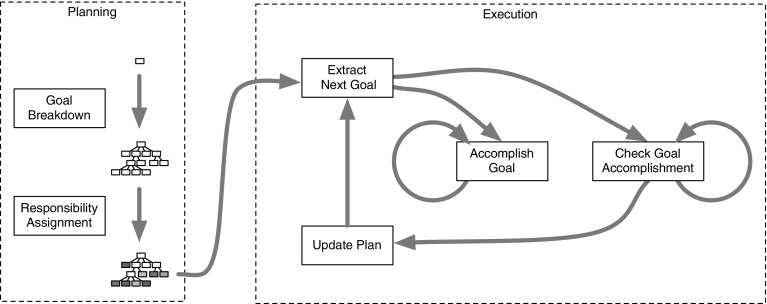
Example task-execution timeline for an assigned responsibility system

**Example task-execution timeline** A task’s accomplishment can be broken down into two stages: planning and execution, as shown in Fig. 5. The planning stage starts with the break down of the job into goals and sub-goals. The goal tests required for the accomplishment testing of these goals are determined. Finally responsibility to satisfy each sub-goal is assigned a LoA. This design makes use of the LoA described in Table 1. The execution stage requires the system to loop through four stages for each of the goals identified in the planning stage: (1) Extracting the goal, which includes selecting the appropriate level of autonomy, setting up the goal test(s), (2) accomplishing the goal, (3) checking the goal’s accomplishment, (4) updating the plan with the new progress information. When the plan is complete, the loop ends.

**Fig. 6:**
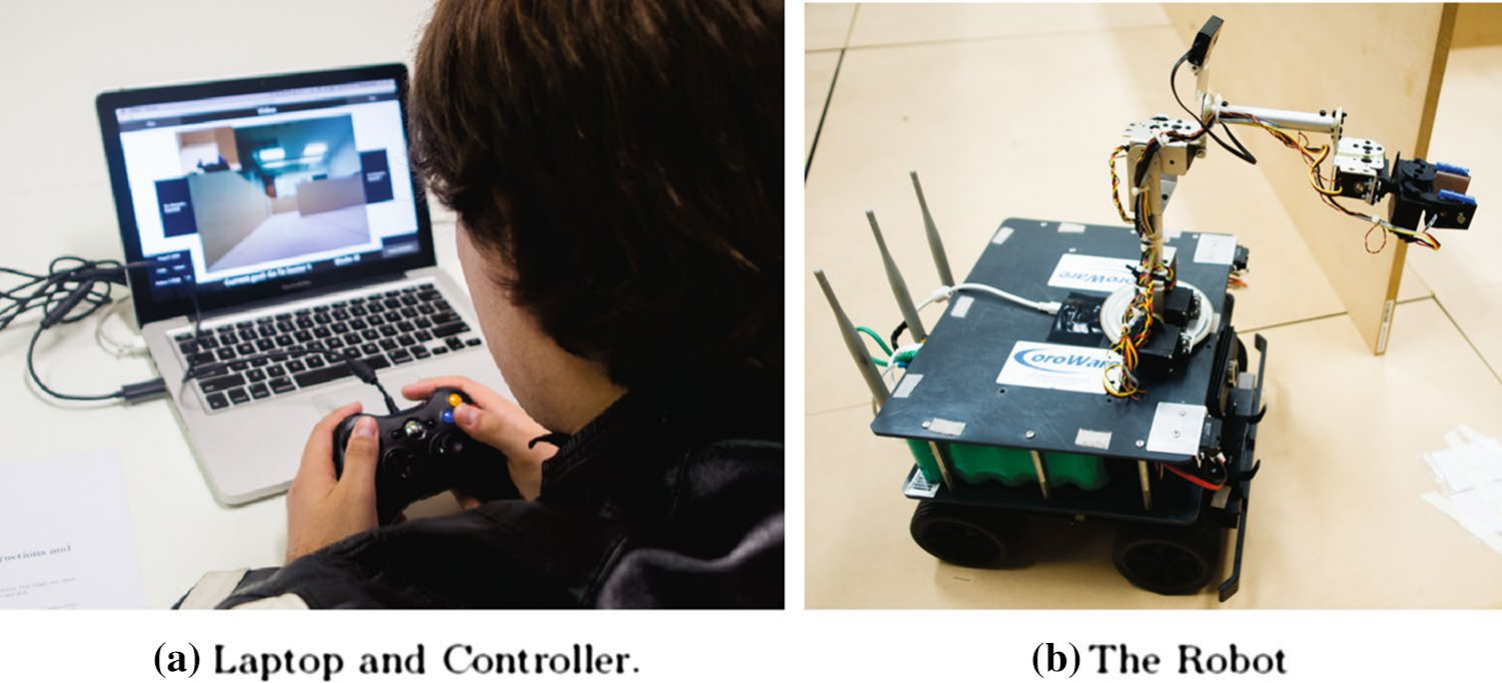
Hardware used in the implementation. Communications between laptop and robot occur through the router mounted on the robot

## Implementation

To evaluate AR experimentally requires human operators to actively use it in a reasonably realistic task. To do this, a prototype implementation of the AR system has been implemented in hardware and software. Because AR is primarily a matter of software, the hardware is mostly off-the-shelf parts, modified to support the requirements of the software. The experiment uses a Coroware mobile field robot, built on top of a Lynxmotion base with four driven wheels, with an Intel D2700MUD Mini-ITX single-board computer running Linux. In contains two cameras, a modified 5 DoF manipulator arm and is equipped with a variety of infrared proximity detectors, force and bump sensors. It is linked via 802.11g wireless to a laptop and Logitech game controller that serve as a teleoperation station.The hardware is distributed across two physical components: the control station (Fig. 6a), and the robot (Fig. 6b). This was chosen because it has the capabilities to fulfil navigation tasks, manipulation tasks, and observation tasks, thus enabling the robot to be used in usability tests of the implemented AR on complex scenarios.

The software was built from scratch for this implementation. It is divided into three modules as pictured in Fig. 4: operation, management, and execution. At any moment, the software must handle input from one or more operators, updates to the plan structure, monitor world states, and execute commands on the robot. This means these modules must function in parallel, somewhat independently of each other. Both this parallelisation and independence mean the system needs a reliable method for communication between modules. All software components were written in the Python programming language (Version 2.7) (Foundation 2017).

The communications between the components of the system are mapped out in Fig. 7. These take two forms, remote object calls, and UDP video streams. To handle object calls between the modules, the Pyro (Python Remote Objects) Python library is used (de Jong 2016).

## Experimental evaluation

**Fig. 7:**
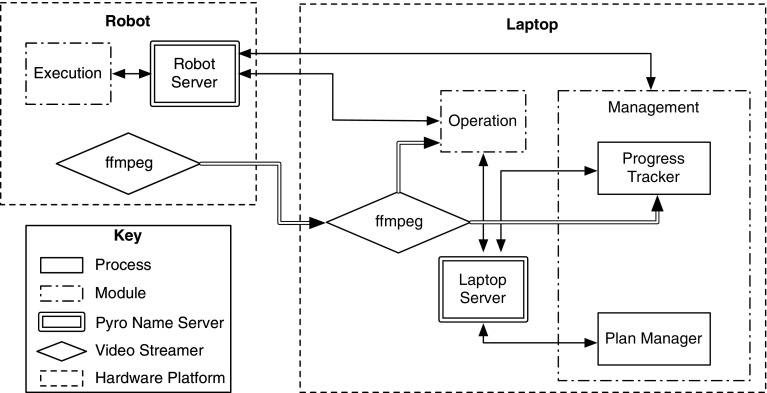
Communications between elements of the system. Single stroke arrows designate remote object calls, double stroke arrows represent video streams

### The experimental task

An end-to-end usability experiment was performed on the implemented teleoperation system to evaluate AR design on operator workload, preferences, effectiveness and efficiency. A repeated measures design was chosen. This is commonly used in user evaluations of interfaces for its efficient use of each participant to test all of the interfaces. The drawback of this design is the possibility for participants to perform better in later trials as they gain experience with the system. This confounding effect can be mitigated by ensuring participants do not undertake trials in the same order. It is important that the sample size (n) in the experiment provides for sufficient power in the inferential statistics to be used (here, multiple ANOVA). This experiment used 20 participants.

**Fig. 8:**
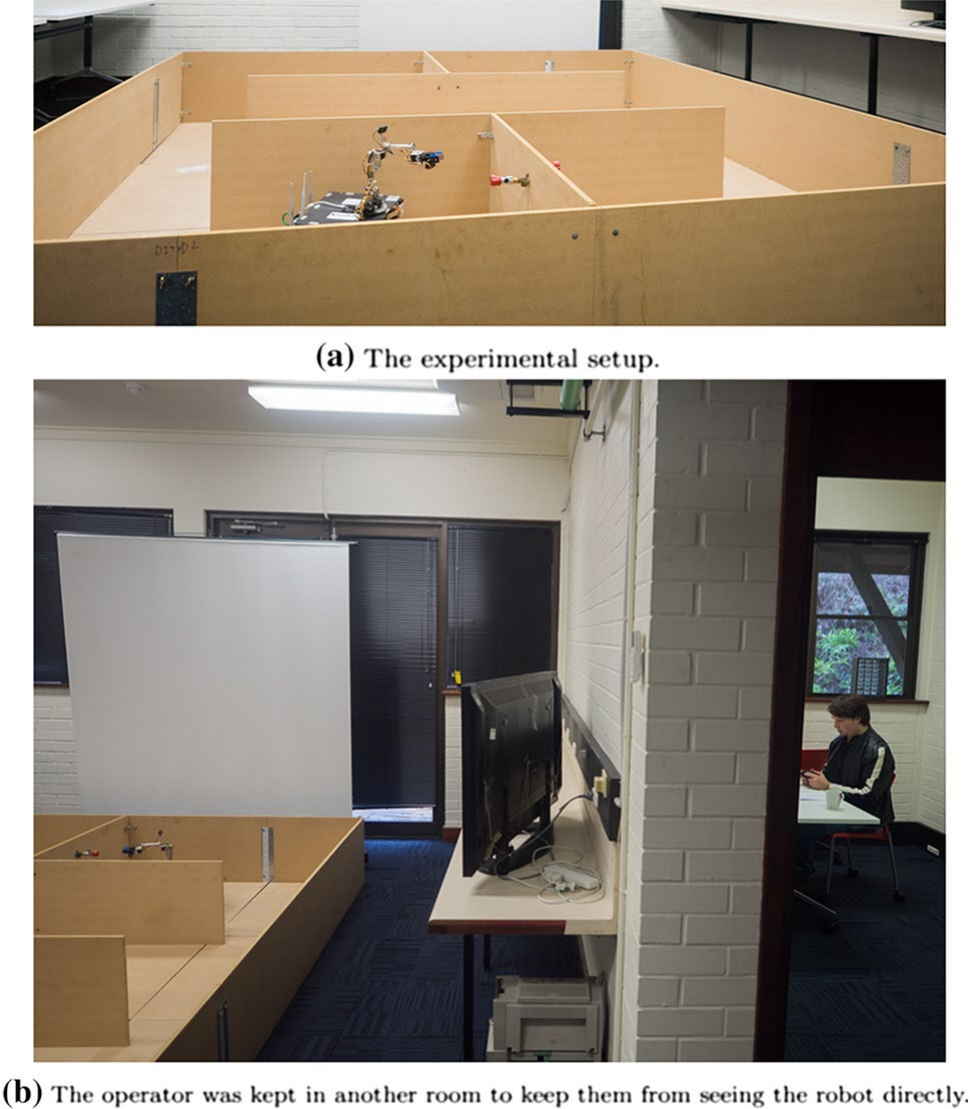
Experimental setup. The robot must be guided through all four sectors, to close all four valves

**Fig. 9:**
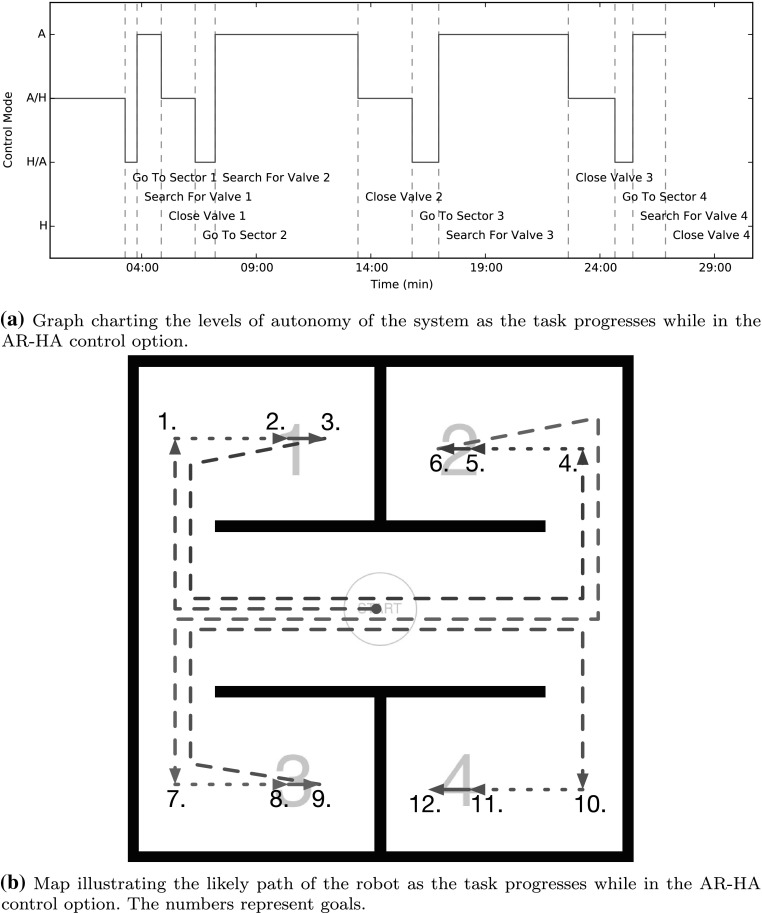
The progress of the assigned responsibility system, illustrating changes in level of autonomy

The task consisted of a mockup maintenance route suggestive of an industrial context. It needed to be complex enough to properly test the human-machine system, yet not so complex as to be too difficult and time consuming for the human operators to learn, and for the automation to accomplish. The robot had to be guided through a sequence of four sectors, each containing a valve that had to be closed using the robot’s arm and gripper. Figure 8 shows the the layout; a sample path is presented in Fig. 9b. The task was broken down into four subgoals: close valve 1, 2, 3, and 4 (the closing order changed every run, and was provided to the operator before each run). Each of these subgoals was further subdivided into three more subgoals, requiring the operator to navigate to the sector, find the valve, and close the valve. When all of the valves had been closed, in the correct order, the task was considered accomplished.

To compare AR to direct, unmediated human control, the experiment included that control mode. The robot had to be operated using four different control options throughout each volunteer’s participation. These control modes covered direct teleoperation (Referred to as control option T) as well as a range of configurations of the AR interface. Control options AR-H and AR-A made use of the progress tracking and plan management sections of the architecture, keeping track of the progress through the task of the robot, as well as keeping the operator informed of that progress. However they had the responsibility assignment for all goals set to H in the case of AR-H and A for AR-A. AR-HA resembles a more typical use of AR as originally imagined, with a set of varying responsibility assignments, as pictured in Fig. 9. The navigation goals were set to A/H, with the human operator in charge of setting waypoints for the automation to execute. The valve finding goals were set to H/A, where the operator had to use the controller to guide the robot’s movements, with the interface providing an overlay highlighting the valve’s location in the camera feed if detected. Finally the valve closing goals were set to full automation (A).

**Fig. 10:**
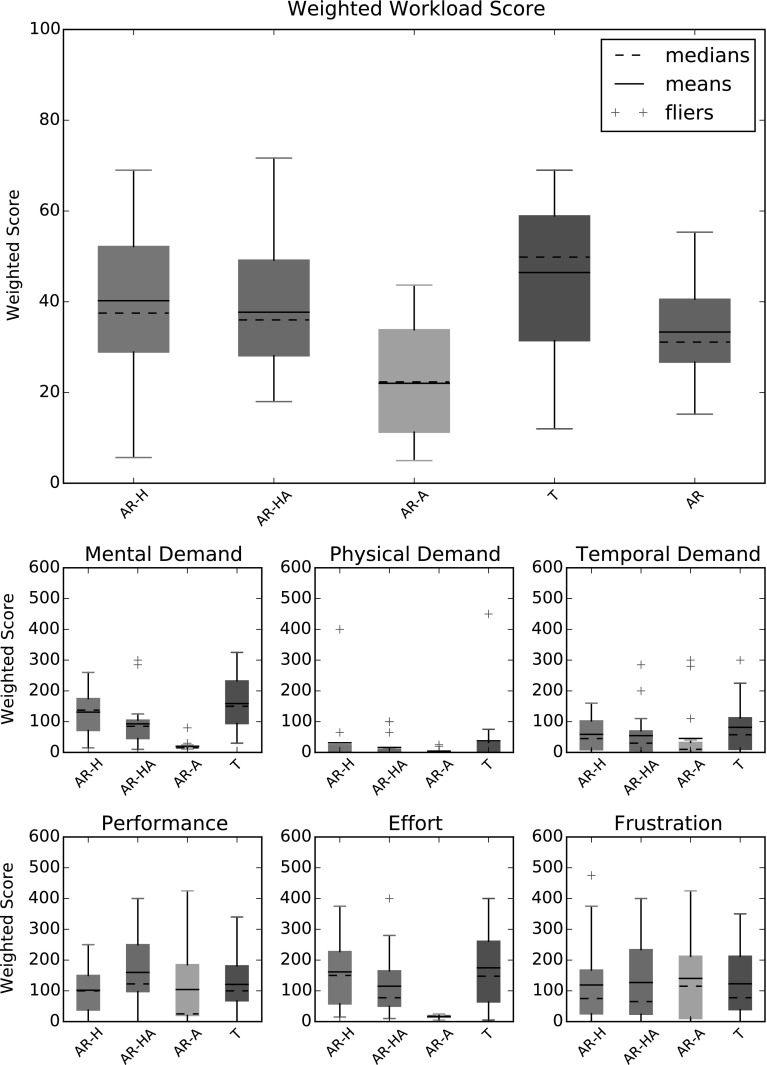
Task load index results for the system. The weighted workload score plot shows the weighted scores for each control option (AR-H, AR-HA, AR-A, T) and for the Assigned Responsibility modes averaged (AR). The smaller plots show the weighted scores given by the participants to each control option for each measure. A higher score means more workload

### Results

During the experiment both qualitative and quantitative data were collected, recording both operator impressions and their performances. Subjective data was collected about the workload placed on the operators in each control option using the NASA Task Load Index (TLX) (Hart and Staveland 1988), as well as operator preferences using a set of informal questions. The TLX combines ratings of mental demand, physical demand, temporal demand, performance, effort, and frustration into an individually Weighted Workload score (WWL). The quantitative data was collected in four sets for each run through the task: one for each valve close goal (consisting of the three subgoals described above). This objective data consisted of measures of effectiveness (i.e. was the valve properly closed?) and efficiency (time taken, collision counts, navigation errors, and manipulation errors occurring during the attempt). The results compare each control condition individually as well as the AR conditions averaged together as an overall representation of what AR offers.

#### Workload

The results of the TLX questionnaire (shown in Fig. 10) showed that when compared option to option, the only control option that was singled out as providing significantly lower workload on the operator is AR-A. Interestingly however, even though AR-A had a lower Weighted Workload (WWL) score than the other options, participants still reported some workload even though they were simply asked to observe the robot perform the task unassisted. This is because not all components of the TLX involve active control movements. While, individually, AR-H and AR-HA were not significantly different to T, they scored slightly lower on average, and the grouped AR options rated significantly lower than T. We may say that real improvements in operator workload are possible, but appear to become important only when the operator is maximally relieved of effort by full automation.

Using work by Grier (2015), it is possible to compare the WWL scores of the modes to the WWL scores of over a thousand systems (from a wide variety of human-machine tasks, from robot control systems and aircraft cockpits), and gain an understanding of how hard the operators were working during the experiment. Over all systems, Grier (2015) reported a mean WWL score of 48.74, higher than the mean of all four control options (AR-H: 40.2, AR-HA: 37.7, AR-A 22, and T: 46.4), making this system, in all configurations slightly less workload intensive than the average task that has been rated using the TLX. This means that the task was not so difficult as to limit the possible observed variance with a ceiling effect. A mean score of 40.2 places the AR-H option near the top 30% of all scores, and 37.7 places AR-HA comfortably between the top 20 and 30%. 22 has AR-A well within the top 10%, while 46.4 places T between 40 and 50%. Grier (2015) categorised the systems, offering an insight into how different types of systems compare, including robot operation systems. Compared to other robot operation systems, all four control options placed in the top 50%, with all three AR options in the top 25%.

#### Preferences

An informal survey collected the participants’ opinion of each control option, having them rank the options on enjoyment. The most enjoyed option was AR-H, with over half of the participants ranking it highest. Participants commented that they enjoyed being in control of the robot, but found the goal accomplishment tracking extremely useful, saving them time and effort when closing the valve. The second most enjoyed option was AR-HA, with those participants citing that this option relieved them of work and was the easiest to use. AR-A and T were both only preferred by one participant each, AR-A because the participant found it required the least work and succeeded in the task, and T because the participant found the automation too unreliable, and would have rather checked the success of tasks manually than to rely on the goal accomplishment tracking.

When the rest of the rankings are taken into account, AR-H still prevailed, followed by AR-HA, followed by T, and with AR-A at the bottom. AR-A was the least enjoyed, with the majority of the participants enjoying being in control, some finding watching the automation struggle a frustrating experience, and finally some stating they felt it was a waste of their time to be there when they couldn’t intervene. This observation is not surprising as it aligns with one of the basic principles of user interface design as proposed by Shneiderman et al. (2009), p. 89, “support internal locus of control”. Collectively, the AR options were found to be more enjoyable than direct teleoperation.

#### Effectiveness

The T and AR-H options caused significantly less goal failures than the AR-HA and AR-A options. In fact there were no goal failures when T was employed, and just the one when AR-H was employed. This goal failure occurring when AR-H was in use was an operator error: an operator forgot to open the gripper after closing a valve, and undid that valve when returning the wrist to a central position. Out of 80 possible such failures, T recorded a 0% failure rate, and AR-H a 1.25% failure rate.

The goal failures recorded during the other two options were caused by the automated sections of the tasks, with a large portion being caused by mapping inaccuracies accumulating and causing the robot to become lost during navigation tasks. AR-HA recorded a 15% failure rate, and AR-A a slightly higher 18.75% failure rate. This clearly shows the vulnerability of an AR system to its weakest automated task, and suggests that either error recovery strategies be implemented to recover from these failures (which was not the case here), or that the LoA of that part of the work be brought back down until the automation is improved. The overall comparison between AR and T shows that collectively, the AR options caused significantly more goal failures, and as stated above this difference is caused by the introduction of automation into the control loop. Altogether, the AR options recorded a 11.67% failure rate.

These data show that out of all the recorded runs, the AR options were less effective than the T option in this case. AR can, however, operate very similarly to direct teleoperation if needed (as was done with the AR-H option) and therefore potentially achieve similar effectiveness levels. This does diminish some of the benefits of AR, but not all, as will be shown later. It is important to note that this experiment can only test this particular implementation of automation, which is not claimed to be indicative of the most up-to-date automation techniques.

#### Efficiency

**Fig. 11:**
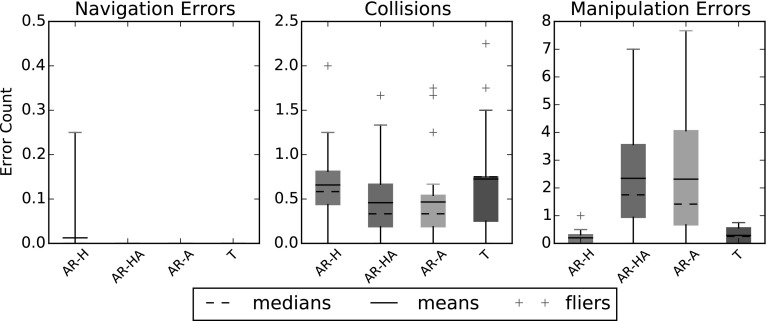
Errors made per valve closed, by type

As shown in Fig. 11, navigation errors were extremely rare, occurring only once during the experiment. Collisions were present in all modes, but occurred more frequently in the manual control modes (AR-H and T). Manipulation errors were far more frequent in the modes that used automation. Both AR-HA and AR-A used the same algorithm to close the valve, so their similar scores are to be expected. While the automated modes made more errors there, they were also capable of making closure attempts at a much faster rate than a human operator, resulting in a higher error count, but a faster task accomplishment rate as discussed below. That speed-accuracy tradeoff could be adjusted fairly easily.

The time data showed that T was significantly slower (236.4 s mean time) than AR-H (180.4 s mean) and AR-HA (174 s mean), which were in turn significantly slower than AR-A (104.7 s mean). This highlights important differences in all of the modes. In AR-A, being fully automated, the system never had to wait for operator input, which had two consequences: a faster task accomplishment rate, and a higher goal failure rate. In AR-HA, the amount of time the robot spent moving would have been fairly similar to that of AR-A, however the automation spent time waiting for waypoints to be set by the human operator, which delayed it.

One of the more surprising differences lies with AR-H and T. With both those options, all of the control task was performed by the human operator. This is reflected in the similar error rates for both options. However, AR-H had a significantly faster accomplishment rate. This difference comes almost entirely from the goal accomplishment tracking during the AR-H runs. With the T option, the operator had to confirm the closure of the valve visually by testing it repeatedly until satisfied. In the AR-H runs, the goal accomplishment tracking was monitoring the strain placed on the wrist by the valve, allowing for a fast detection of the valve’s state, cutting down the number of grasp attempts needed to close it, and thus saving time.

These two sets of data tell a different story, with T outclassing AR as a whole on errors made, but causing a significant increase in time taken for task accomplishment. Overall, the AR system resulted in faster task accomplishment across the board (153 s mean), with the ability to trade a higher error rate for more speed or vice versa (by switching from human control to (semi-automated control) if needed, making it more efficient than the direct teleoperation option.

## Observations and future work

The experiments shown in this paper showed that AR is a viable form of robot teleoperation, capable of accomplishing the tasks set out to operators while imposing less workload on these operators when compared to traditional teleoperation approaches. However, this study can only hint at the situations where AR should be deployed over other adjustable autonomy approaches, and several questions remain to be answered. Are planned changes always easier on the human operators? Are automated changes a viable option when they could catch the human operators unaware? Are manual changes just another task human operators would rather avoid? Under what circumstances should the assignment contract be violated in the interests of safety or efficiency?

### On automated changes

It became apparent during the trials conducted for this research, that even when planned, automated changes in levels of autonomy could be an issue for human operators. During the trials, one goal was to line up the robot with a valve, a goal which was set to the H/A LoA (human control with assistance from the automation). The automation assisted by highlighting the valve in the video feed to indicate to the operator that the robot was detecting it. The next goal was to close the valve, in full automation. When the valve was detected by the progress tracker, the goal would change, and the automation would take over.

This drastic change in level of autonomy, even if planned, caused a lot of frustration to the operators, as the goal might be considered accomplished before they were happy with the placement of the robot. Once the automation took over, the human operator was locked out of the control loop, adding to that frustration if the automation was struggling with closing of the valve due to a poor start position. This proved to be so frustrating that some operators tried to hide the valve from the robot’s camera until they were satisfied with their position. A potential solution to this kind of problem would be to make the operator one of the “sensors” for the goal accomplishment tracking, allowing them to report their satisfaction with the robot’s state when ready. Here is an example of the consideration must be paid to the triggers of automated changes in LoA (planned or not) to avoid inadvertently increasing operator workload.

### On error recovery

In case of errors, or unforeseen changes in the environment, it is likely that the plan as designed before the task started will no longer produce the desired results. Therefore, a strategy for handling divergences from this plan must be decided on. This problem was not addressed in this paper directly, as it dealt only with the core concepts of AR, but potential solutions can be imagined and discussed here. Plan-altering situations can be considered to fit either of two categories: (1) smaller problems that require a correction before the plan can be resumed, and (2) major problems that will prevent the plan from ever completing. An example of the first would be the loss of a tool required for a future goal in the plan. Before the plan can be completed, the tool must either be found again, or another acquired. Once either has happened, the plan can resume from where it left off. A major issue would be the destruction of a piece of equipment that the plan required the robot to repair. The equipment can now no longer be repaired, rendering the plan un-accomplishable.

It is likely that the major failures would result in a cancellation of the plan rather than any kind of in-task re-planning. More interestingly, the smaller problems require on-the-spot inclusion of additional steps (or the replacement of current steps), and thus the modification of the existing plan. Who makes these changes is an important question, as keeping operators aware of the plan is a requirement for AR. A low-tech solution could be to give full control of the robot to the human operator when such a problem arises, with the express goal of returning the situation to an acceptable state. Yet this defeats some of the benefits of AR by imposing unexpected tasks on the human operator, and forcing a single control mode to be used. To make use of the full capabilities of AR, some amount of local re-planning must be done, either automatically or manually. This re-planning needs to add goals, and assign them a LoA. Once the re-planning is done, the modifications must be made available to the operator for approval, ensuring all of the actors in the system are aware of their responsibilities before the task resumes. It is possible that for common problems a library of patches to the plan could be created, which could be rapidly deployed when these problems arise. This could somewhat ameliorate the problem of great additional operator workload, as these patches would likely be familiar to operators that use the system often.

## Conclusion

While full automation of the robots used today in teleoperated tasks may be desirable, it is in many cases currently infeasible. In the immediate future, a mix of automation and manual control seems the most promising avenue for useful robot control in most scenarios. Direct control teleoperation still reigns, as it is hard to beat the reliability of human operators in most scenarios. If automation is still too untrustworthy to be charged with taking over control, the introduction of automation should primarily focus on assisting the human operators. Let automation take over the small simple tasks that still require time and effort from a human operator, freeing them spend more time and effort on more important tasks such as strategic planning and problem-handling. As time passes and automation techniques become more refined and reliable, more tasks can be automated, gradually reducing demands on the human operator.

Assigned Responsibility was designed in part to help this process of gradual automation. The breakdown of the overall task into goals and subgoals sets delimitations that can be used to limit the influence of automation to the simple tasks without jeopardising the greater plan. This also provides context for automation research, breaking down a complex task into a series of simpler tasks, that can be automated one by one instead of as a whole. While that is happening, the robot can keep on working, with the non-automated parts of the plan handled by a human operator. This paper has demonstrated that an Assigned Responsibility system can be used to successfully control a robot through a task using a variety of configurations. It is hoped that this contribution will support further work in the skilful mixing of automation and human-based control of remote robots toward optimal performance.

## Footnotes

1 This is task and implementation specific, but mostly means a self-contained piece of work that can be accomplishable using a single process. The goal of closing a door could be broken down into two subgoals, navigating to the door and closing the door for example. It could also be broken down into more subgoals, if needed. At some stage however, it is likely that overly small subgoals will frustrate human operators, who will resent having to accomplish baby steps. A balance between the needs of automation and the wants of human operators will have to be struck, possibly on a task to task basis
